# Effect of different durations of compression therapy on quality of life and recurrence after thermal ablation for varicose veins: a protocol for a prospective, multicenter, randomized controlled trial

**DOI:** 10.3389/fmed.2026.1744286

**Published:** 2026-03-13

**Authors:** Lin Yang, Zhang Zhang, Chunmin Li, Ruipeng Zhang, Liang Wang, Jiehua Qiu, Ye Tian

**Affiliations:** 1 Department of Vascular Surgery, The First Affiliated Hospital of Xi’an Jiaotong University, Xi’an, China; 2 Department of Vascular Surgery, The Second Affiliated Hospital of Air Army Medical University, Xi’an, China; 3 Department of Vascular Surgery, ChaoYang Hospital of Capital Medical University, Beijing, China; 4 Department of Vascular Surgery, Shaanxi Provincial Peoples’ Hospital, Xi’an, China; 5 Department of Interventional Vascular Surgery, Dongguan People’s Hospital, Dongguan, China; 6 Department of Vascular Surgery, The Second Affiliated Hospital of Nanchang University, Nanchang, China; 7 Department of Vascular Surgery, The First Affiliated Hospital of Xinjiang Medical University, Urumqi, China

**Keywords:** compression stockings, compression therapy, protocol, quality of life, randomized controlled trial, recurrence, thermal ablation, varicose veins

## Abstract

**Background:**

Compression therapy is routinely recommended after the thermal ablation of varicose veins (VVs), but the optimal duration of treatment remains controversial. International guidelines acknowledge the lack of evidence to support a specific duration of compression therapy (grade 2C). In current clinical practice, the selection of therapy options (1–3 months or even longer) rely mainly on the clinical judgment of physicians, with significant variability in patient compliance and quality of life. This trial aims to determine the effects of different compression durations (1, 3, and 6 months) on recurrence, quality of life, and complications at 12 months after surgery.

**Methods:**

This will be a prospective, multicenter, randomized, parallel-group trial. This study will include 600 patients with great saphenous vein insufficiency (C2–C6) treated with endovenous radiofrequency or laser ablation at 7 high-volume centers. The participants will be randomly assigned to the following groups at a 1:1:1 ratio: the one-month (OM) group, which will wear class II compression stockings (23–32 mmHg) for 1 month after surgery; the three-month (TM) group, which will wear compression stockings for 3 months; and the six-month (SM) group, which will wear compression stockings for 6 months. All the groups will wear the same compression stockings and follow the same instructions for wearing the stockings. The primary outcome is the recurrence of VVs at 12 months; the secondary outcomes include the closure rate of the great saphenous vein, disease-specific quality of life assessed by the Aberdeen Varicose Vein Questionnaire (AVVQ), the Chronic Lower Limb Venous Insufficiency Questionnaire (CIVIQ) score, the Venous Clinical Severity Score (VCSS), the incidence of postoperative complications (pain, ecchymosis, paresthesia, deep vein thrombosis, etc.), and patient compliance. The follow-up times are 1, 3, 6, and 12 months after surgery.

**Discussion:**

This trial fills a key gap in evidence concerning the optimal duration of compression therapy after endovenous thermal ablation and the effect of providing optimal compression therapy on recurrence. The results of this study will provide high-level evidence for a standardized duration of compression therapy and contribute to updated future clinical guidelines.

**Clinical trial registration:**

chictr.org.cn, identifier ChiCTR2100049550.

## Introduction

Varicose veins (VVs) of the lower extremities affect 15%–20% of adults worldwide and can lead to serious complications, such as pain, edema, skin damage, and ulcers (1, 2). Endovenous thermal ablation procedures [radiofrequency ablation (RFA), endovenous laser ablation (EVLA)] are recommended as first-line therapy options (3–5). Postoperative compression therapy using elastic stockings is considered the essential treatment for VVs, which can reduce postoperative pain, edema, and bruising and may prevent the postoperative recanalization of VVs (6, 7). Although compression therapy is recommended by guidelines as a necessary treatment after thermal ablation, the optimal duration of compression therapy remains controversial, and strong evidence is lacking (8–10). Recent guidelines have only clarified the necessity of compression therapy, but still not specify the optimal duration of compression therapy (5). Therefore, there is considerable variability in the use of compression therapy after the thermal ablation of VVs among different centers and regions: some centers recommend 1 month of compression therapy after thermal ablation, whereas others recommend 3 months or longer (11–13). Long-term compression therapy is often recommended, especially for patients with venous ulcers (C5-6). It is undeniable that the long-term use of compression stockings can result in leg discomfort and skin irritation, which could lead to poor compliance in patients who use compression therapy for a long period (14, 15), especially in early stage with C2-3. Therefore, recent studies have focused mainly on short-term (several days/week) regimens and the short-term results of compression therapy (16–18), including parameters such as immediate pain relief and return to work after surgery. A recent study reported that there was no difference in the great saphenous vein (GSV) closure rate in patients who received short-term compression therapy during a 6 month follow-up (10). Owing to the lack of long-term follow-up data, evaluating the effects of different durations of compression therapy on the postoperative recurrence of varices and the quality of life (QoL) of patients is very difficult. Additionally, owing to hemodynamic abnormalities such as GSV and deep venous reflux, short-term compression therapy may have limited effects on patients’ QoL scores and symptom improvement. Thus, we believe that determining the optimal duration of compression therapy is crucial for the evaluation of patient-centered efficacy (QoL scores and long-term recurrence), and few high-quality randomized controlled trials (RCTs) have evaluated the medium- and long-term outcomes of compression therapy after surgery in patients with VVs (10). The latest clinical guidelines confirm that the optimal duration of compression therapy after the thermal ablation of VVs is still based on clinical judgment rather than evidence-based medical data (5). This multicenter RCT aims to evaluate the differences in VV recurrence rates and QoL scores at 12 months after thermal ablation surgery among three compression therapy strategies and provide new evidence for the optimal duration of compression therapy after thermal ablation.

### Objectives

The primary objective of this study is to determine the recurrence of VVS at 12 month after thermal ablation in patients with different durations of compressure therapy, assessed by duplex ultrasound scanning and clinical physical examination. The secondary objectives are the saphenous vein (GSV) closure rate at 12 month, the closure failure is defined by a segment of the reopen vein ≥5 cm by ultrasound. The VCSS, AVVQ, CIVIQ scores are compared among the three groups at baseline, 1, 3, 6, and 12 month, as well as the pain scores [using the Visual Analog Scale (VAS)] at baseline, 24 h, and 1 month after surgery. Postoperative complications are recorded in three groups, and the compliance of compressure therapy is investigated.

## Methods

The SPIRIT reporting guidelines were followed in the development of this protocol (19).

### Study design

**Figure 1:**
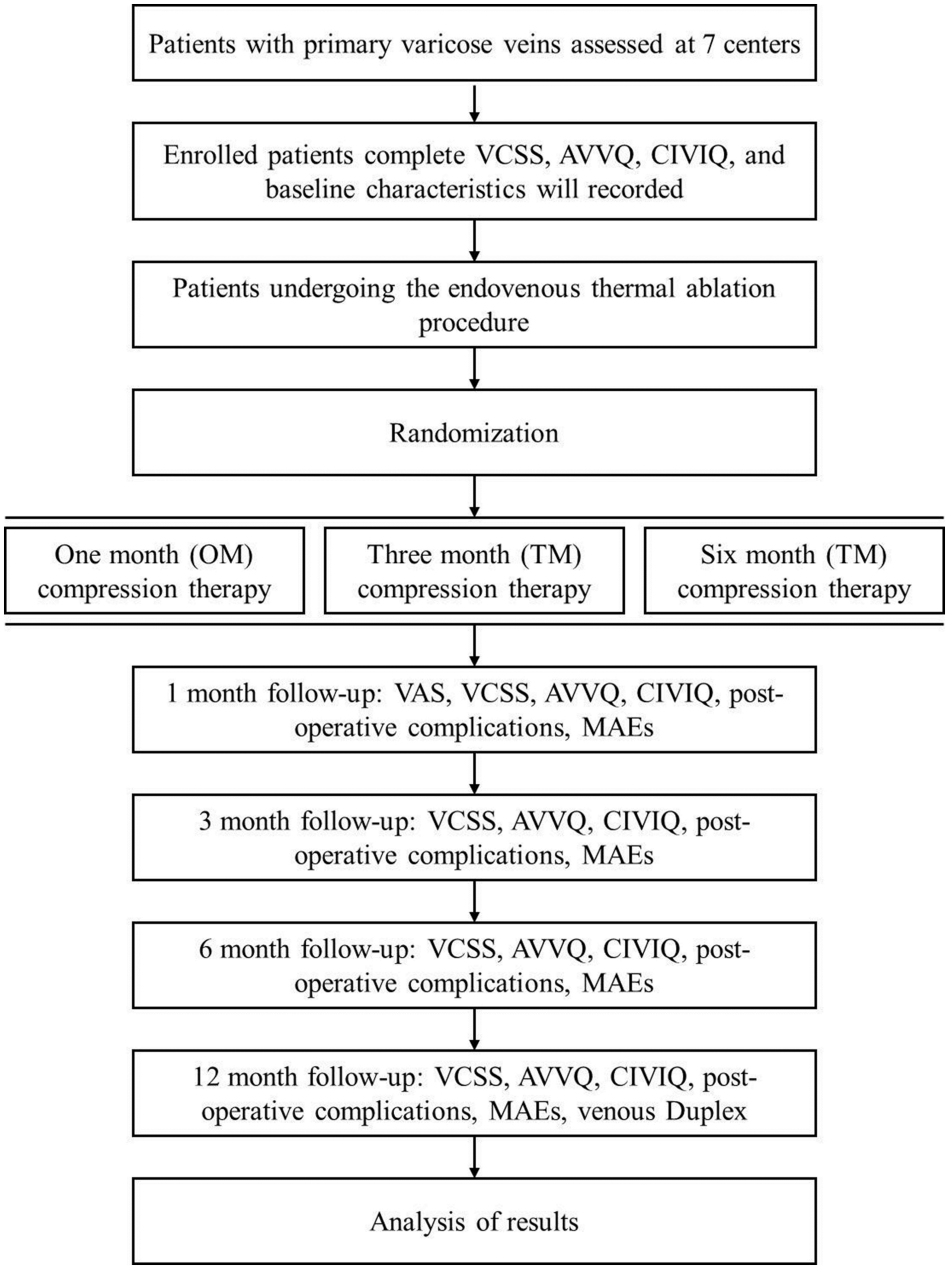
The trial flowchart. VCSS, Venous Clinical Severity Score; AVVQ, Aberdeen Varicose Vein Questionnaire; CIVIQ, Chronic Lower Limb Venous Insufficiency Questionnaire; OM, one-month; TM, three-month; SM, six-month; VAS, Visual Analog Scale.

This will be a prospective, multicenter, open-label, randomized, three-arm trial with a 12 month follow-up (Figure 1). The SPIRIT checklist is provided in Supplementary File S1.

### Study setting

Seven high-volume vascular surgery centers in China will participate in this trial:

The First Affiliated Hospital of Xi’an Jiaotong University (Coordinating Center).
Shaanxi Provincial People’s Hospital.
The First Affiliated Hospital of Xinjiang Medical University.
Beijing Chaoyang Hospital, Capital Medical University.
Tangdu Hospital, Air Force Medical University.
Dongguan People’s Hospital, Dongguan, China.
The Second Affiliated Hospital of Nanchang University, Nanchang, China.

### Participants

Patients with VVs of the lower extremities will be included in this trial, all patients will diagnosed via clinical examination and duplex ultrasound examination at all centers, and all patients will sign an informed consent form.

### Inclusion criteria

An age ranging from 18 to 70 years.
The presence of primary, symptomatic unilateral or bilateral lower limb VVs (CEAPs C2-C6).
A GSV reflux duration >0.5 s on duplex ultrasound.
A GSV diameter of 3–12 mm (thigh segment).
Scheduled to undergo RFA or EVLA (1,470 nm laser) of the GSV.
The ability and willingness to provide informed consent.

### Exclusion criteria

A history of ipsilateral VV intervention (surgery, ablation, or sclerotherapy).
A history of deep vein thrombosis (DVT) or post-thrombotic syndrome (PTS).
The presence of significant peripheral arterial disease (ABI < 0.8).
Severe infection of the treatment site.
A known allergy to compression stocking material;
The presence of severe medical comorbidities precluding surgery/follow-up (e.g., uncontrolled cardiac failure, terminal illness).
Pregnancy or lactation.
Participation in another conflicting clinical trial.
The inability to comply with compression therapy or follow-up schedule.

### Randomization and blinding

Eligible, consenting patients will be randomized at a 1:1:1 ratio to one of the following groups: the one-month group (OM group, elastic stockings for 1 month), three-month group (TM group, elastic stockings for 3 months), and six-month group (SM group, elastic stockings for 6 months) using a central, computer-generated, stratified block randomization sequence via a secure randomization-generated web-based system. Patients and physicians cannot be blinded owing to the nature of the intervention (duration visible to the patients). The outcome assessors (Duplex ultrasound technicians, QoL questionnaire administrators) and statisticians will be blinded to group allocation.

### Interventions

#### Thermal ablation

All participants will undergo standardized RFA (ClosureFast™, Medtronic) or EVLA (Eufoton 1,470 nm ring laser Biolitec) of the incompetent GSV under ultrasound guidance and tumescent anesthesia (20), which will be performed by surgeons with experience in performing >200 ablation procedures. In brief, the GSV trunk was cannulated just below the knee under ultrasound guidance, and then radiofrequency catheter (ClosureFast, Medtronic, United States) was inserted 2 cm below the saphenofemoral junction and ablated at a temperature of 120 °C for 20 s per segment. The same technique was used in the EVLA group by using a radial laser fiber (1,470 nm, Eufoton, Italy) at power: 10 W and energy: 70 J/cm. The VVs below the knee was treated by using ultrasound-guided foam sclerotherapy and a pin stripping (mini-phlebectomy) procedure.

#### Postoperation compression

All participants will have identical Class II (23–32 mmHg) full-length graduated compression stockings applied in the operating room immediately after ablation, and the patients will be asked to wear the stockings during the day and night for 48 h and then during waking hours (following the protocol). The durations of compression therapy will be as follows:

OM group: Wear stockings during waking hours for 1 month after surgery.
TM group: Wear stockings during waking hours for 3 months after surgery.
SM group: Wear stockings during waking hours for 6 months after surgery.
Compliance will be monitored via patient diaries and questionnaires.

### Primary outcome

The primary outcome is the recurrence of target VVs at 12 months (21, 22). New recurrence is defined as the postoperative appearance of VVs that did not exist in the VV area in the lower limbs before surgery; primary recurrence is defined as the reappearance of VVs that existed in the VV area before surgery during follow-up. Both types of recurrence are defined as postoperative recurrence.

### Secondary outcomes

The secondary outcomes are as follows.

#### Closure rate of target veins

The target vein occlusion rate at 12 months is defined as ultrasound-confirmed recanalization of the treated GSV segment (≥5 cm), and the occlusion rate will be calculated as the number of target venous closure cases/total number of cases.

#### QoL assessment

Disease-specific QoL at baseline and at 1, 3, 6, and 12 months after surgery will be measured by the AVVQ (total scores ranging from 0–100, with 100 indicating the worst QoL) (23).

#### Venous Clinical Severity Score (VCSS)

All patients will be asked to complete the VCSS questionnaire to assess the severity of venous disease features at baseline and at 1, 3, 6, and 12 months after surgery. The VCSS questionnaire consists of 10 questions, each scored as 0 (none), 1 (mild), 2 (moderate), or 3 (severe), with a total score ranging from 0 to 30, with 30 representing the most severe venous disease (24).

#### Chronic Lower Limb Venous Insufficiency Questionnaire (CIVIQ)

The CIVIQ is an internationally recognized test for measuring the QoL of patients with chronic venous disease, 11, 12 which consists of 20 questions comprising four QoL domains: the pain (4 items), physical (4 items), social (3 items) and psychological (9 items) domains. Each question receives a score of 1–3 points (1 for mild and 5 for severe), and the overall score range from 0 to 100 points. These clinical studies reported only the overall outcome, 13 which indicates a patient’s QoL (24, 25).

#### Postoperative pain

The Visual Analog Scale (VAS, 0–10, 0 for no pain and 10 for the worst pain imaginable) will be used for the assessment of postoperative pain at 24 h and 30 days (26).

#### Postoperative complications

Postoperative complications (within 30 days) will include hematoma, ecchymosis, paresthesia, skin burns/paresthesia, phlebitis, infection, and DVT.

#### Major adverse events (MAEs)

MAEs are defined as myocardial infarction (MI), stroke, and all-cause death occurring during the postoperative follow-up period.

#### Trial duration

**Figure 2:**
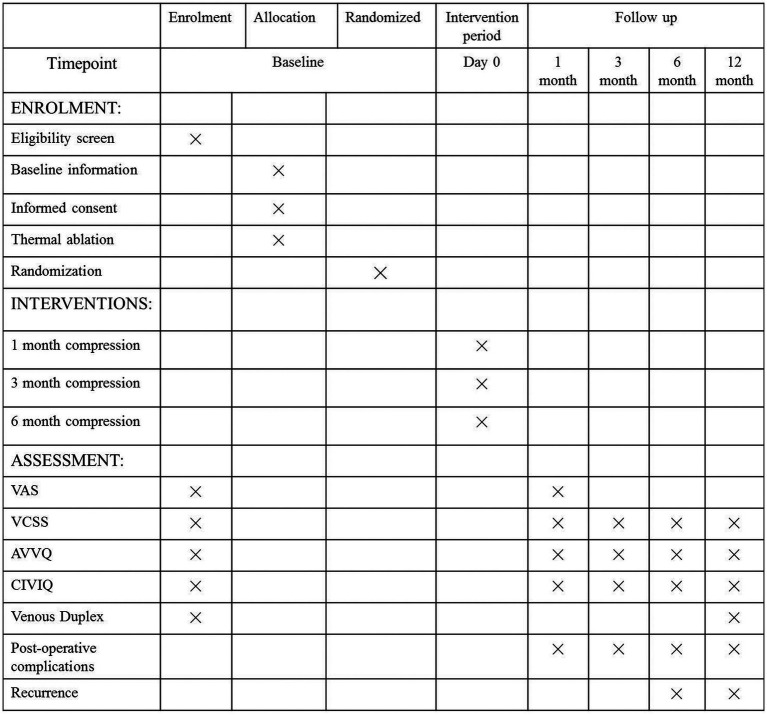
The trial protocol: The baseline and follow up questionnaires of this trials, including the schedule of enrolment, interventions, and assessments. VAS, Visual Analog Scale; VCSS, Venous Clinical Severity Score; AVVQ, Aberdeen Varicose Vein Questionnaire; CIVIQ, Chronic Lower Limb Venous Insufficiency Questionnaire.

On the basis of the experimental standard criteria, the case distribution of each central hospital, and previous similar trials, this study is expected to be completed in approximately 24–36 months (Figure 2; Supplementary File S1).

### Baseline

Each research center will have a fixed medical staff (doctors or nurses from the surgical team) that is responsible for collecting information and conducting follow-up visits, one researcher who is responsible for collecting information, and another researcher who is responsible for assessing the quality of the questionnaire. All researchers will receive unified training before formal follow-up. At baseline, we will collect main demographic data, surgery-related data, and perioperative complication data from each patient and complete the collection of questionnaire data.

### Follow-up

The researcher will assist each patient in outpatient follow-up and data collection at 1, 3, 6, and 12 months (Figure 1). For patients who cannot visit the outpatient clinic for follow-up, follow-up will be completed by video telephone and social media software, and schedule a specific time for the ultrasound examination.

### Sample size and study duration

According to previous studies (27–32) and recent systematic analyses, the 12 month recurrence rate after the thermal ablation of VVs in the lower extremities is approximately 3%–5%. The setting in the OM group is 4% and the non-inferiority threshold *δ* is −6% (17). With a statistical power of 90% and a significant equivalence of 1.7% (one-sided), at least 511 patients (170 in each group) needed to be recruited. However, considering the potential withdrawal rate of 20% and the very low incidence of adverse events in this trial, the total sample size was increased to 600 patients (200 in each group). The patients will be followed up for 12 months, and the study is expected to last for 36 months.

### Recruitment

Patient recruitment will be conducted among inpatients at each center; patients who meet the inclusion criteria will be informed of the study and receive relevant information. Potential patients will be further examined by the researcher (doctor), and eligible patients will be identified after medical history collection and ultrasound examination. Each participating patient will be informed about the trial process, treatment methods, risks, compensation, and follow-up. Finally, the patient or their guardian will provide informed consent.

### Statistical analysis

Analysis will follow the intention-to-treat (ITT) and per-protocol (PP) principles. Continuous outcomes (AVVQ score, VCSS, VAS score, and CIVIQ) will be analyzed using linear mixed models (LMMs) adjusted for baseline values, center, CEAP class, and time. Non-inferiority will be declared for the AVVQ score at 12 months if the upper limit of the 95% CI for the difference (OM-SM or TM-SM) is <*δ* (2.0 points). Recurrence rates will be compared using Kaplan-Meier survival analysis and Cox proportional hazards models. Complications will be compared using chi-square tests or Fisher’s exact tests. Compliance will be summarized descriptively. Subgroup analyses (e.g., by CEAP class, ablation type) are planned. Sensitivity analyses will be performed to assess the impact of missing data. SPSS version 21.0 will be used. *p* < 0.05 will be considered to indicate significance for superiority analyses.

### Data collection and confidentiality

Trained researchers will collect data by using a unified case report form. Patient demographic data, laboratory results, and surgery-related data will be extracted from the electronic medical records system, and baseline CEAP classification, VCSS, AVVQ, EQ-5D scores and ultrasound data will be recorded. All the data will be cross-checked by two independent researchers to ensure accuracy. To reduce the loss rate and ensure that each participant completes the follow-up on time, we will record each participant’s mobile phone number and social media account (WeChat), and the researchers will remind the participants of the follow up before each follow-up time point. Moreover, we will ask each participant about their compliance with compression therapy to assess whether they interrupt or deviate from the intervention plan. The participants will receive a separate trial identification number on the basis of the order of inclusion. All information collected in this study, including electronic and paper questionnaire data, will be securely stored in each center for at least 10 years. All data will be strictly confidential and accessible only to trial team members, and anonymous trial data will be shared with other researchers upon reasonable request.

### Data monitoring and quality control

The ethics committee of each center will regularly review the conduct of the trial. The trial steering group and independent data monitoring committee (composed of biostatisticians, surgeons, investigators and assistants) will meet regularly throughout the trial to review the conduct of the trial. Major changes such as sample size adjustment or inclusion criteria will be reported to the monitoring committee, whereas non-major changes will be recorded only by the investigator. The Ethics Committee and the Monitoring Committee will jointly modify or suspend the study.

### Availability of data and materials

All data analyses and manuscripts will be available from the corresponding author upon request (for valid reasons).

### Withdrawal and termination

Patients can withdraw from the study for any valid reason, but we will not encourage patients to withdraw from the study voluntarily, and we are prepared to address any adverse events. The study will be terminated if any of the following situations occur: (a) one treatment is significantly superior or inferior to the other treatment; or (b) a serious adverse event occurs.

### Ethical approval and trial registration

This study was approved by the Ethical Committee of the First Affiliated Hospital Xi’an Jiaotong University (Approval No. XJTU1AF2021LSk-099). Written informed consent will be obtained from all participants or guardians. This trial was registered on the chictr.org.cn website.^1^ The study will follow the recommendations for physicians involved in research on human subjects adopted by the 18th World Medical Assembly, the 1964 Helsinki declaration, and later revisions.

## Discussion

Compression therapy (elastic bandages or stockings) is an essential therapeutic measure after the thermal ablation of VVs in the lower limbs. Compression therapy can immediately promote venous occlusion and reduce patient pain. Recent studies have focused on short-term follow-up after compression therapy and the effects on short-term perioperative effects (postoperative pain, return to normal work and QoL scores). No larger RCT has focused on the effect of compression therapy on the recurrence of VVs and QoL scores in the medium and long term after surgery (follow-up greater than 12 months), only a very small sample size study provided the limited information (31). Although long-term compression therapy may cause limb discomfort in patients, determining the optimal duration of compression therapy to evaluate the medium- and long-term effects after thermal ablation is crucial, especially to evaluate the effect on QoL. This trial aims to address a basic unresolved question in the management of VVs after thermal ablation: the optimal duration of compression therapy. This will be a large multicenter RCT that aims to generate high-level evidence to optimize clinical care for patients by directly comparing the three most common durations of compression therapy (1, 3, and 6 months). The results of the study will be used to directly update national and international clinical practice guidelines.

## Ethics and dissemination

### Ethical considerations

This study was approved by the Ethical Committee of the First Affiliated Hospital Xi’an Jiaotong University (Approval No. XJTU1AF2021LSk-099). Written informed consent will be obtained from all participants or guardians. This trial was registered on the chictr.org.cn website (see text footnote 1 respectively). The study will follow the recommendations for physicians involved in research on human subjects adopted by the 18th World Medical Assembly, the 1964 Helsinki declaration, and later revisions. To protect the participants’ privacy, this study will not collect any sensitive information, such as race, sexual orientation, or religious beliefs. All data will be anonymized at the time of collection. Throughout the research process, the research team is committed to adhering to the highest ethical standards and legal compliance principles.

### Safety considerations

There are no significant health related risks associated with our proposed study. Adverse events (AEs) are unexpected medical events that occur in patients and may or may not be related to a study device. Regardless of whether AEs occur, the AE form should be completed and sent to the principal investigator by email within 24 h. If the principal investigator considers an event to be “study related” (i.e., caused by the implementation of any study procedure), the AE will be further reported to the research ethics committee. Investigators at each center should report any AE according to the requirements of their research ethics committee and the sponsor.

If any damage related to the pressure therapy study occurs during the study, the fund will bear the corresponding treatment costs and provide compensation in accordance with national laws and regulations.

### Dissemination plan

The results of this study will be published in peer-reviewed journals and presented at national or international conferences. The datasets analyzed during this study and the research protocol is available upon request from the corresponding author.

## Footnotes

1
https://www.chictr.org.cn/showproj.html?proj=120121
